# Physical communication pathways in bacteria: an extra layer to quorum sensing

**DOI:** 10.1007/s12551-025-01290-1

**Published:** 2025-03-04

**Authors:** Virgilio de la Viuda, Javier Buceta, Iago Grobas

**Affiliations:** Theoretical and Computational Systems Biology Program, Institute for Integrative Systems Biology (I2sysbio), CSIC-UV, Catedrático Agustín Escardino Benlloch 9, 46980 Paterna, Spain

**Keywords:** Bacterial communication, Physical stimuli, Bacterial signaling, Bacterial sensing, Environmental adaptation, Emerging properties

## Abstract

Bacterial communication is essential for survival, adaptation, and collective behavior. While chemical signaling, such as quorum sensing, has been extensively studied, physical cues play a significant role in bacterial interactions. This review explores the diverse range of physical stimuli, including mechanical forces, electromagnetic fields, temperature, acoustic vibrations, and light that bacteria may experience with their environment and within a community. By integrating these diverse communication pathways, bacteria can coordinate their activities and adapt to changing environmental conditions. Furthermore, we discuss how these physical stimuli modulate bacterial growth, lifestyle, motility, and biofilm formation. By understanding the underlying mechanisms, we can develop innovative strategies to combat bacterial infections and optimize industrial processes.

## Introduction

Bacterial communication serves as a link between the intracellular space (cytosol) and the extracellular environment. By exchanging information with their surroundings, bacteria can sense environmental conditions and adapt accordingly (Singhi and Srivastava 2020). The scope of bacterial communication is vast, ranging from solitary cells interacting with their immediate environment to intricate signaling networks within densely populated colonies (Jiang et al. 2016). Importantly, these individual and collective modes of communication are not independent but interact and influence one another.

Traditionally, studies of bacterial communication have focused on quorum sensing and biochemical pathways (Moreno-Gámez et al. 2023). Quorum sensing enables microorganisms to communicate chemically by responding coordinately to the accumulation of extracellular chemical signals (autoinducers) and reprogramming gene expression as a function of cell density (Ábrahám et al. 2024). Synthetic biology has leveraged this as a tool for programming gene expression patterns in microbial communities (Weber and Buceta 2016; Simpson et al. 2023). However, the effectiveness of these signals can be significantly influenced by the surrounding environment (Tabraiz et al. 2021). For example, fluid flow has been demonstrated to impede quorum sensing by washing away molecular messengers (Kim et al. 2016), and pH and temperature can degrade crucial quorum-sensing molecules (Boyer and Wisniewski-Dyé 2009).To ensure effective signaling, bacteria must produce sufficient quantities of autoinducers to surpass the detection thresholds of their neighbors while minimizing noise in the signaling process (Weber and Buceta 2011, 2013).

From an evolutionary perspective, the inherent limitations of chemical signaling have likely driven the development of alternative communication strategies in bacteria, including physical signals such as mechanical, electrical, and light-based signals (Humphries et al. 2017; Tessaro et al. 2019; Copenhagen et al. 2021; Manna et al. 2020; Tong 2024). Physical signals are less dependent on the surrounding medium and can propagate through mechanisms beyond simple diffusion, allowing them to travel greater distances and facilitate faster communication (Reguera 2011). Furthermore, physical stimuli often interact with and complement quorum sensing, creating a feedback loop that enriches and diversifies bacterial communication systems (Bavaharan and Skilbeck 2022). This interplay results in a more diverse and resilient communication system, enhancing the overall adaptability and responsiveness of bacterial cells (Belas and Suvanasuthi 2005; Gode-Potratz et al. 2011; Li et al. 2012; Gayán et al. 2017; Genova et al. 2019; Gordon and Wang 2019; Harper and Hernandez 2020).

This literature review focuses on how bacteria perceive and exchange physical stimuli with their environment and the effects of these interactions on their metabolism, growth, and development. We will first examine the key physical stimuli bacteria may exchange with their environment and how researchers have used such stimuli in recent studies. Then, we will focus on how bacteria use these physical magnitudes to interact with each other and to coordinate collective behaviors. Finally, we will highlight how physical stimuli can shape colony development, summarizing their potential to enhance antimicrobial treatments and bioindustrial applications.

## Physical stimuli between bacteria and their environment

### Mechanical stimuli and mechanosensing

The ability of bacteria to sense the mechanical properties of their growth media allows enables them to adapt to environmental changes (Gordon and Wang 2019), optimize their growth (Wang et al. 2023a, b), and decide whether to choose a planktonic state or to form biofilms (Santore 2022). Recent studies have revealed that bacteria possess sophisticated mechanical response mechanisms, allowing them to detect and respond to substrate stiffness, surface topography, and mechanical stress (Gordon and Wang 2019).

**Fig. 1:**
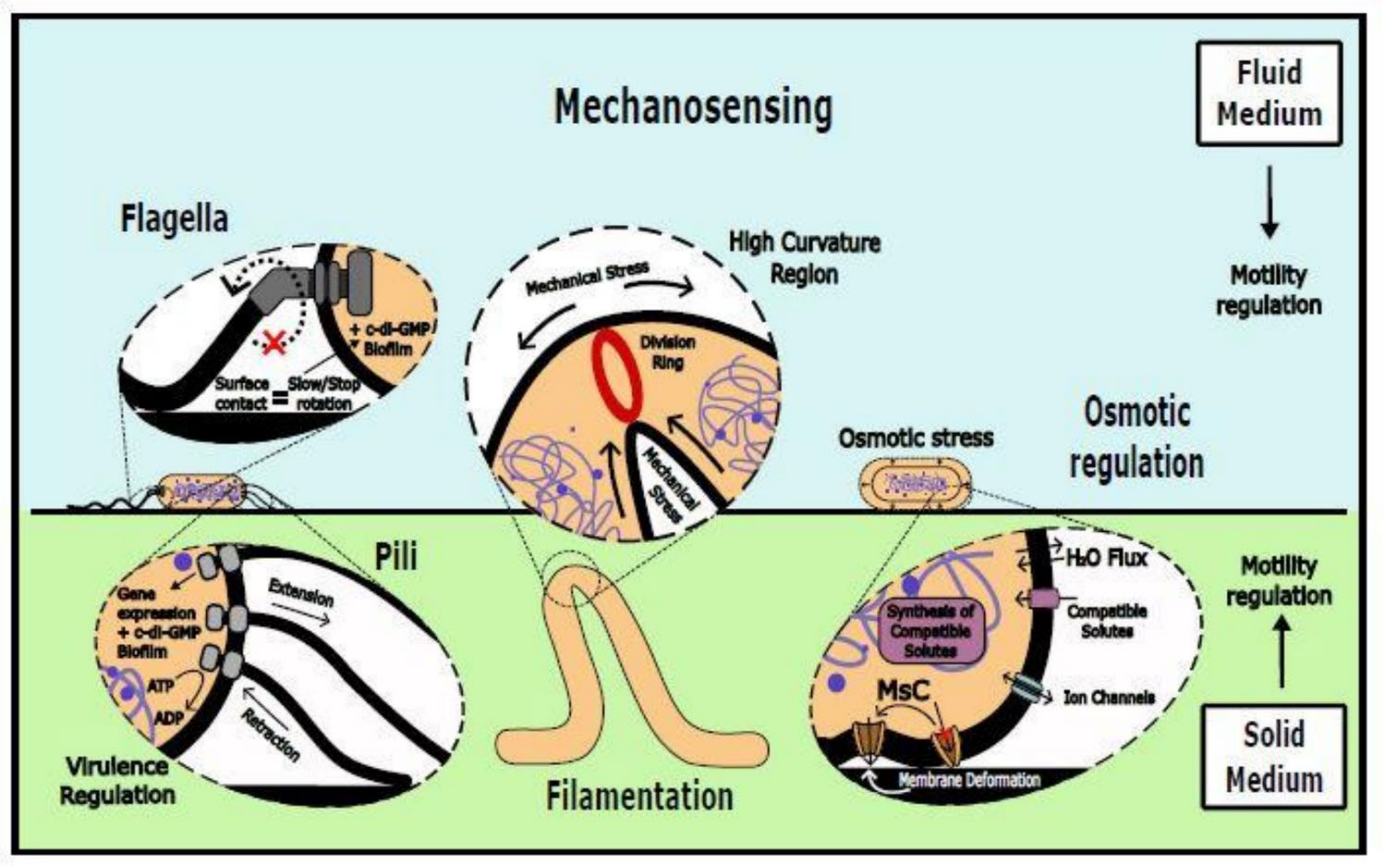
Mechanosensing. Bacteria can sense mechanical cues, such as surface stiffness or liquid media viscosity, and respond actively through flagella and pili or passively through mechanosensitive channels. Mechanical strain in the bacterial membrane affects intracellular protein localization and divisome dynamics. Osmotic pressure, resulting from differences in solute concentrations with the environment, can also induce mechanical stress on the bacterial membrane

Individual cells can sense the physical properties of the media through appendages that are also important for their motility, such as pili or flagella (Fig. 1) (Gordon and Wang 2019; Hershey 2021). Surface sensing through these appendages may regulate adhesion, biofilm formation, and virulence (Persat et al. 2015; Inclan et al. 2016; Mordue et al. 2021). Sensing becomes relevant during colonization since the stiffness of the substrates that bacteria colonize can range from ultrasoft (dermal fillers have elastic moduli 0.02–3 kPa and living tissues 0.2–30 kPa) to hard (orthopaedic implants have elastic moduli 5–300 GPa) (Wang et al. 2016; Guimarães et al. 2020). A well-studied example is *Escherichia coli*, which can respond to shear forces through the FimH adhesive subunit of type I pili (Sauer et al. 2016; Laventie and Jenal 2020). This leads to increased bacterial adhesion to surfaces (Thomas et al. 2002; Evstigneeva et al. 2021), a critical advantage during urinary tract infections, where FimH transitions to a high-affinity binding mode to resist urine flow (Sauer et al. 2016). In *P. aeruginosa*, the extension and retraction of Type 4 Pili (TFP) actively regulates virulence-related genes for a range of substrate stiffness of 10 to 100 kPa (Koch et al. 2022). Interestingly, this stiffness range matches many human tissues: lung, spleen, thyroid, muscle, and skin (Guimarães et al. 2020; Koch et al. 2022). It has also been shown that the cell-surface-exposed protein PilY1 is an active mechanosensor that transduces mechanical changes in the bacterial envelope into varying intracellular levels of cyclic-di-GMP (c-di-GMP), a crucial second messenger in bacterial signaling (Ryan 2013; Valentini and Filloux 2016; Zhan et al. 2024). This finding aligns with research that showed that shear applied by external fluid flows, combined with friction-like adhesion to the surface, might act as mechanical cues that increase c-di-GMP production in *P. aeruginosa* (Rodesney et al. 2017). Nonetheless, this view has been challenged by the fact that the drag force bacteria experience when they swim (~ 1 pN) is higher than what most flow cell experiments exert onto the cells (Chawla et al. 2020). In the gram-positive model organism *Bacillus subtilis*, increasing levels of shear stress enhanced enzyme production and increased reactive oxygen species (Sahoo et al. 2003).

As c-di-GMP is tightly related to biofilm formation, substrate stiffness strongly impacts early microcolony development in *P. aeruginosa* (Gomez et al. 2023). The transition from motile to sessile lifestyles could be driven by surface contact and subsequent attachment (O’Toole 2003; Joo and Otto 2012; Hershey 2021; Santore 2022). However, it has been suggested that this initiation of the biofilm lifestyle can also be caused by attachment and aggregation among different bacteria (Thomas et al. 2002). Biofilms exhibit distinct gene expression, metabolism, and physical properties compared to their planktonic counterparts (Wang et al. 2015; Flemming et al. 2016; Zappa et al. 2023).

These mechanical properties of the media are crucial as they will decide whether bacteria will remain still (hard substrates) or whether they will move, and how they will do so (Kearns 2010; Gordon and Wang 2019; Grobas et al. 2020). For example, liquid media induces swimming motility powered by flagella, which consists of roughly straight runs followed by random directional changes (tumbles) (Wadhwa and Berg 2022). On viscoelastic surfaces, bacteria swarm, a collective motion where colonies expand as droplets with reduced surface tension due to surfactant production (Kearns 2010). During swarming, cell rafts undergo swirling-like motion powered by the flagella of the cells within the colony (Srinivasan et al. 2019; Jeckel et al. 2019; Be’er et al. 2020; Kotian et al. 2020; Kasallis et al. 2023). Alternatively, bacteria can also use pili to regulate a different kind of motion called twitching (Mattick 2002), which depends on the substrate stiffness: a change from 33.7 to 573 kPa led to a rise in the twitching speed of *Pseudomonas aeruginosa* from 0.06 to 0.18 µm/min (table S3 in Cont et al. 2023).

The cell envelope is another key player in bacterial mechanosensing (Gordon and Wang 2019). Recent findings from our group demonstrate that increased mechanical stress due to bending in *E. coli* filaments changes the preferred sites for bacteria cell division through a diffusion-based passive mechanism (Nadal et al. 2025). This is supported by positive feedback loops between high curvature regions (or high bending strain) and protein accumulation (Fig. 1) (Rombouts et al. 2023). Another study in single *E. coli* cells (Genova et al. 2019) describes how bacteria exposed to stepwise increases in Pressure axial difference (ΔP) exhibit elongation and narrowing, resulting in reduced cell volume. Both analytical and finite-element models suggest that extrusion loading generates axial tensile stress while reducing hoop (transverse) tensile stress, depending on ΔP magnitude. This volume reduction during extrusion raises internal cell pressure, likely due to increasing osmolarity as water exits the cytoplasm. These volume and pressure passive changes illustrate the critical role of water fluxes in maintaining equilibrium between the cytoplasm and the external environment.

The water fluxes needed to equilibrate substances between the cytoplasm and the environment have been demonstrated to affect cell volume, turgor pressure, cell wall strain, and cytoplasmic membrane tension. These water fluxes, or osmosis, are a mechanism to regulate uncharged solute, salt ion, and biopolymer concentrations (Wood 2015). Bacteria employ multiple mechanisms to sense and respond to osmotic stress and avoid dehydration or rupture, categorized into three groups. Firstly, typically present in microorganisms are Mechanosensitive channels (Msc), proteic pores in the membrane that detect transmembrane mechanical stress (Fig. 1). They function as emergency release valves during hypoosmotic stress, passively opening on a millisecond time scale (Boer et al. 2011; Rasmussen and Rasmussen 2018). Secondly, solute transporters that import or release organic osmolytes called compatible solutes. They work while the cell releases K + maintaining physiologically appropriate levels of cellular hydration and turgor (Fig. 1). Some examples are membrane transporters like ProX, OpuAC, and BetP act as sensors and transporters for the compatible solute glycine betaine in *E. coli* (Schiefner et al. 2004), *B. subtilis* (Horn et al. 2006), and *C. glutamicum* (Perez et al. 2014), respectively (Bremer and Krämer 2019). Thirdly, the active signal transduction mechanism to respond to external stimuli: two-component systems (TCS). Generally, a TCS comprises a sensor protein (histidine kinase or HK) and its corresponding response regulator (RR). The enterobacterial TCS PhoQ/PhoP can phosphorylate upon osmotic upshifts, between other stimuli, due to perturbation in membrane thickness and lateral pressure in the cytoplasmic membrane (Yuan et al. 2017). In *Salmonella typhimurium*, the PhoQ/PhoP system promotes virulence in host environments as a response (Groisman et al. 1992, Groisman 2001). Still, *E. coli* promotes acid and heat tolerance (Han et al. 2023). Usually, as each particular TCS is specialized to respond to a specific environmental signal (e.g., pH, nutrient levels, osmotic pressure, redox state, quorum-sensing proteins, and antibiotics), multiple TCSs may be present in a single bacterial cell (Tiwari et al. 2017). Interestingly, the LytS/LytR TCS in *Staphylococcus aureus* controls cell death and lysis by sensing perturbations in the membrane’s electrical potential (Patel and Golemi-Kotra 2015).

### Electric stimuli

**Fig. 2:**
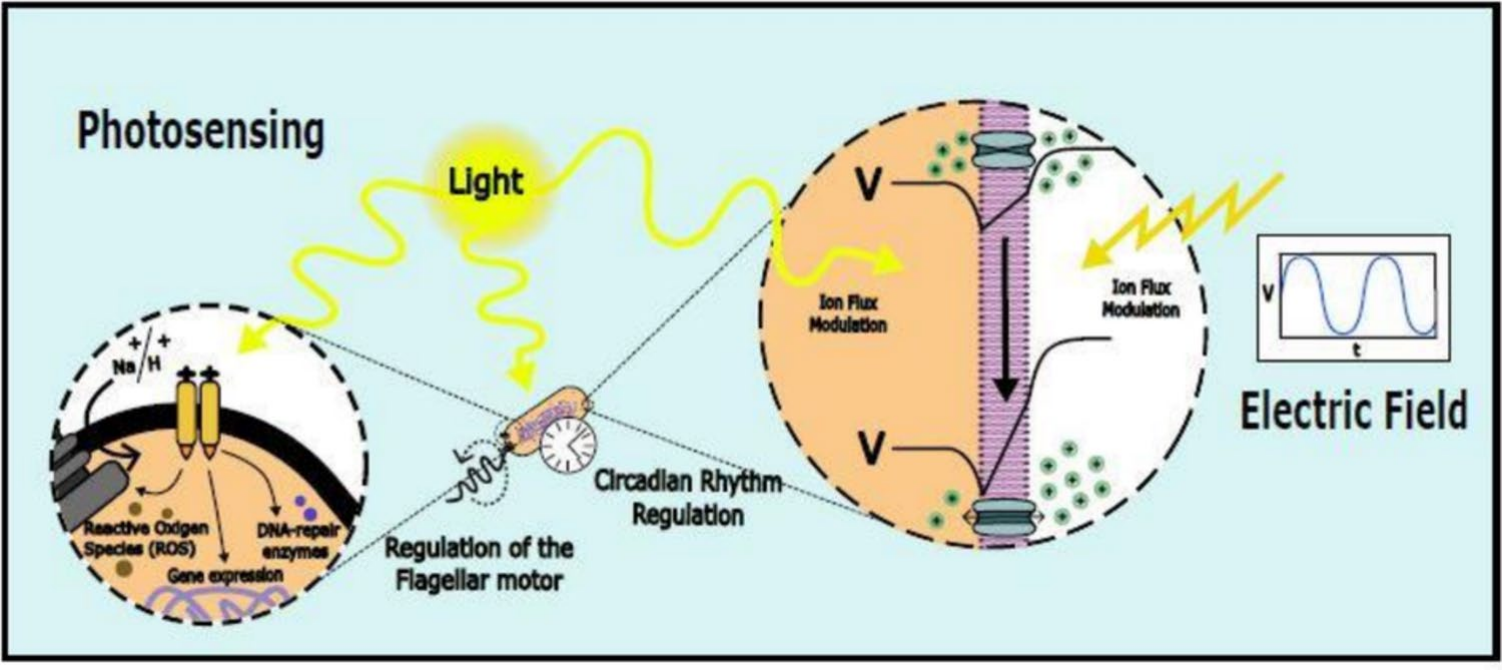
Electric Stimuli and photosensing. Electric fields can influence bacterial ion transport and consequently, membrane potential, affecting key processes such as flagellar motility. Exposure to light can lead to oxidative stress, but light-sensitive proteins (rhodopsins, bacteriophytochromes, light-oxygen-voltage (LOV) domains, etc.) also regulate motility, gene expression, DNA repair, or synchronize activities with circadian rhythms (e. g., cryptochromes)

A gradient of substances, particularly ions, across the bacterial cell membrane generates an electric potential tightly regulated by membrane ion pumps. This potential is essential for energy generation, powering flagellar motility, nutrient exchange, and intercellular interactions (Fig. 2) (Benarroch and Asally 2020; Lo et al. 2024). Membrane potential has also been linked to antibiotic susceptibility. A study on *E. coli* exposed to ampicillin revealed that cells could be classified as “killed,” “persisters,” or “viable but non-culturable” based on their membrane potential (higher to lower membrane potential, respectively) (Jin et al. 2023). This agrees with other studies in *B. subtilis* that found that hyperpolarized cells had ~ 3 × lower chance of survival under spectinomycin treatment (Lee et al. 2019). It was also shown that the treatment itself with ribosome-targeting antibiotics led to a ~ 2 h long hyperpolarization in both *B. subtilis* and *E. coli* due to cation flux, linking the membrane potential to the ability of bacteria to cope with antibiotic stress (Lee et al. 2019; Bruni and Kralj 2020).

Beyond their endogenous bioelectric properties, bacteria also respond to externally applied electric fields, which have been explored as tools for studying bacterial physiology and viability. When a microorganism is placed in a pulsed electric field, the cell membrane undergoes structural changes such as electroporation (Zimmermann 1986) or electroosmosis (Castro et al. 1993). At high voltages (3.5 V/µm for 90 µs), the *E. coli* membrane ruptures, altering its permeability—a process exploited for sterilization (Tao et al. 2015; Beretta et al. 2019). Weaker electric fields (60 mV/µm for 2.5 s) have been applied to determine the viability of *B. subtilis* and *E. coli* cells, reinforcing the critical role of membrane potential in bacterial stress responses (Stratford et al. 2019). Complementary studies demonstrate that live *E. coli* has higher cytoplasmic conductivity (1.5 S/m) than dead cells (1.4 S/m), corresponding to an 8.22% decrease upon cell death (Li et al. 2018). This is attributed to membrane compromise in dead cells, confirmed by increased GFP diffusivity (FRAP) and nanoparticle permeability assays showing ion and macromolecule leakage. Thus, the measured conductance of dead cells was indistinguishable from the surrounding medium, indicating that their cytoplasmic ionic composition had equilibrated with the external environment. This correlates with membrane potential collapse, as membrane potential is fundamentally maintained by the asymmetric distribution of ions across the membrane.

Changes in the ion gradient across the membrane also affect ATP metabolism (Benarroch and Asally 2020; Lo et al. 2024), therefore changing the rotation speed of bacteria flagella (Fig. 2) (Fung and Berg 1995; Krasnopeeva et al. 2019; Biquet-Bisquert et al. 2024). Many studies have used this as a reliable real time meter of this electrical property (Gabel and Berg 2003; Krasnopeeva et al. 2019; Biquet-Bisquert et al. 2024). Disruptors of membrane potential, such as CCCP, have also been used in *E. coli* and *B. subtilis* to prove the importance of membrane potential in cellular processes. For example, adding CCCP to these bacteria led to a spatial delocalization of cytoskeletal and cell division proteins (Strahl and Hamoen 2010).

Mechanotransduction mechanisms and light may also affect membrane potential dynamics (Fig. 2). The latter will be discussed in the following sections. It has been demonstrated that calcium transients and, consequently, membrane potential spikes, in *E. coli* immobilized on glass in a minimal medium were reduced compared with cells immobilized on glass under an agarose pad of the same medium (Bruni et al. 2017). Similarly, in *Vibrio cholerae*, the passive stoppage of the flagellum upon surface contact induces a transient hyperpolarization of the bacterial cell membrane, which initiates biofilm formation (Van Dellen et al. 2008). Thus, dissipation of the membrane potential through valinomycin blocks the transition from transient to permanent attachment, which prevents the initiation of *V. cholerae* biofilms.

### Photosensing

Bacteria possess sophisticated mechanisms to detect and respond to environmental light through specialized photosensory proteins. These proteins—rhodopsins, phytochromes, xanthopsins, cryptochromes, phototropins, and blue light-using flavin (BLUF) domains—enable bacteria to interpret light as a critical environmental signal (van der Horst and Hellingwerf 2004). While initially thought to be unique to phototrophic bacteria, recent findings reveal that light sensors, such as bacteriophytochromes in *Deinococcus radiodurans* and *P. aeruginosa*, also function in non-phototrophic species (Maresca et al. 2019), underscoring the widespread role of light in bacterial adaptation (Gomelsky and Hoff 2011; Wilde and Mullineaux 2017).

Each type of photoreceptor responds to specific light wavelengths, initiating signaling pathways that influence bacterial behavior and survival (Wilde and Mullineaux 2017). Rhodopsins contribute directly to energy generation, while bacteriophytochromes and light-oxygen-voltage (LOV) domains in species like *B. subtilis* regulate gene expression and motility (Fig. 2) (Maresca et al. 2019; Tuttobene et al. 2020). Some receptors, like cryptochromes, help synchronize bacterial activities with daily light cycles (Fig. 2), which is critical for adapting to fluctuating environments (Maresca et al. 2019). These sensory capabilities are ecologically advantageous. For instance, soil bacteria rely on bacteriophytochromes to prepare for water stress by initiating protective responses at sunrise, when overnight moisture begins to evaporate (Hatfield et al. 2023).

Photosensing supports a range of adaptive responses. For instance, bacteria may produce protective pigments or activate DNA repair mechanisms under harmful light (Wilde and Mullineaux 2017; Liu et al. 2019a, b). Blue light can produce reactive oxygen species (ROS), damaging cellular components, which has implications for bacterial growth and survival (Tuttobene et al. 2020). Photosensing even plays a role in pathogenesis, with light-responsive LOV domains in *Brucella abortus* enhancing virulence by promoting replication in host macrophages (Tuttobene et al. 2020). Light can also prompt gene expression changes, optimizing metabolic functions and communal behaviors, such as biofilm formation (Karlsson et al. 2023). Light has also been used to encode “memory” in bacterial communities. Specific cells within these communities exhibit transient membrane potential spikes. It has been observed that cells that have experienced such spikes are more likely to do so again in the future (Larkin et al. 2018). This has been exploited in *B. subtilis* biofilms to encode membrane-potential-based memory by irradiating cells for 5 s with 438 nm light (Yang et al. 2020). Similarly, the light of 470 nm in conjunction with the molecule Ziapin has been used to manipulate the membrane potential of individual *B. subtilis* cells (de Souza‐Guerreiro et al. 2023). Ziapin drives modulation of the membrane potential via an optomechanical effect by changing the membrane capacitance and triggering the opening of the chloride channel (de Souza‐Guerreiro et al. 2023). Another example of photo-electrotransduction in bacteria corresponds to the bacterial rhodopsins. They function as light-driven proton pumps (del Rosario et al. 2010) (Proteorhodopsin, Bacteriorhodopsin), ion channels (Channelrhodopsins), or also as chloride ion pumps (Halorhodopsin), contributing to the electrochemical gradient and modulating directly the membrane potential (Klare et al. 2008).

Blue light has also been shown to create membrane damage and slow down flagellar motion, which has implications for motile bacteria (Krasnopeeva et al. 2019; Han 2023). For example, blue light slows down cells in swirling motion during swarming motility in *B. subtilis* (Grobas et al. 2021), and red, green, blue, and white light exposure resulted in a decrease in the swarm diameter indicating that light reduced the ability to swarm (Fessia et al. 2024).

These properties have notable biotechnological applications: photoreceptors such as the cyanobacterial CcaS-CcaR TCS are now used to create light-responsive regulatory tools for industrial processes like fermentation (Tabor et al. 2011; Baumschlager and Khammash 2021; Hoffman et al. 2022). In this way, light has been used to guide bacteria and induce pattern formation in the form of letters, popular movie scenes, and portraits of famous characters (Arlt et al. 2018; Frangipane et al. 2018; Lugagne et al. 2024).

### Magnetic field

**Fig. 3:**
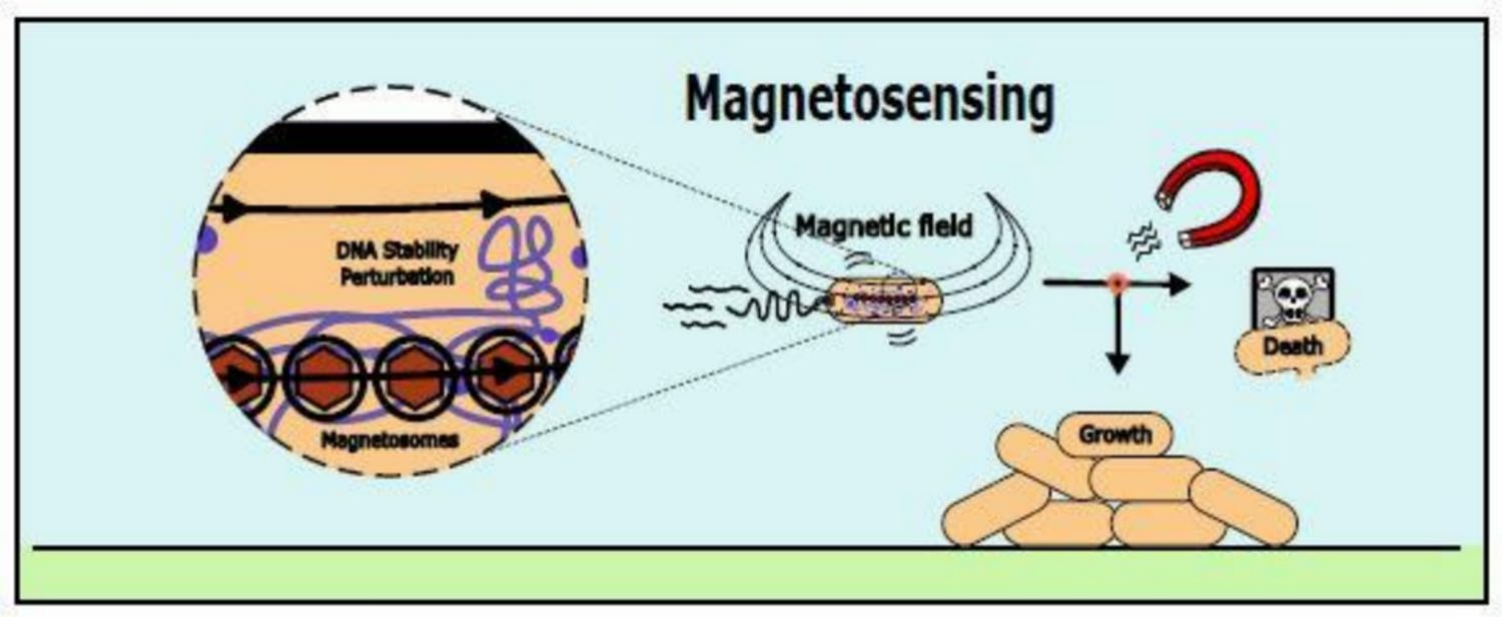
Magnetic Sensing. Some bacteria biomineralize magnetosomes, membrane-enclosed magnetic nanoparticles whose alignment guides bacteria in magnetic fields and directs their motility in one dimension. Magnetic fields can also impact bacterial growth and DNA stability, affecting metabolism, gene expression, and morphology

Some aquatic bacteria possess the ability to sense magnetic fields through the biomineralization of magnetosomes (Barber-Zucker and Zarivach 2017), membrane-enclosed magnetic nanoparticles whose alignment guides bacteria, passively, in magnetic fields (Fig. 3) (Lefèvre and Bazylinski 2013). The main ecological advantage of this magnetotactic bacteria (MTB) is their ability to settle in low-oxygen zones (Smith et al. 2006; Faivre and Schüler 2008) since their motion is not random but it is guided by their dipole moment, sufficiently large to sense the geomagnetic field (Frankel and Blakemore 1980). This reduction in directional freedom enhances the efficiency of aerotaxis, allowing them to locate optimal environments more effectively (Smith et al. 2006). Researchers have harnessed this magnetic guidance to direct MTB to specific locations, offering promising applications such as drug delivery for disease treatment (González et al. 2014; Kotakadi et al. 2022).

The effects of magnetic fields on bacterial behavior and physiology extend beyond MTB (Liu et al. 2024a, b, c). Research indicates that various types of magnetic fields influence bacterial growth, metabolism, gene expression, and morphology, often in species-specific ways (Fig. 3) (Horiuchi et al. 2001; Beretta et al. 2019; Jabłońska et al. 2022). For instance, sinusoidal magnetic fields (30 mT, 50 Hz) stimulate growth in *E. coli* and *S. aureus* during the exponential growth phase (Nawrotek et al. 2014). On the contrary, Ahmed et al. 2013 showed a decrease in the growth rate of *S. aureus* for a wide range of intensities (0.5 to 2.5 mT) and frequencies (3–500 Hz). Additionally, static fields exert diverse effects: Potenza et al. (2004) found enhanced *E. coli* colony formation under a 300 mT field, while Horiuchi et al. (2002) reported a 105-fold increase in cell count in high magnetostatic conditions (5.2–6.1 T). Somehow, there is not a consensus about the impact of magnetic fields on bacteria fitness (Buchachenko 2016; Jabłońska et al. 2022).

Beyond growth effects, magnetic fields can influence bacterial metabolism. In *Rhodococcus erythropolis*, a 50 mT field enhanced phenol degradation across concentrations of 0.3–1.2 g/L (Pospíšilová et al. 2015). High-intensity fields, such as 14.1 T, have been shown to alter gene expression in *Shewanella oneidensis* (Gao et al. 2005), while weaker fields of 0.1 to 1 mT (50 Hz) have been shown to modulate gene expression and cell morphology in *E. coli* (Cellini et al. 2008). Mechanistically, magnetic fields may affect DNA stability via increased oxidative stress (Li and Chow 2001; Naskar et al. 2021), which induces hydroxyl radicals and amplifies the effects of nitrogen oxide on membrane proteins. Furthermore, magnetic fields also affect the interactions of essential ions like Mg^2^⁺, Ca^2^⁺, and Zn^2^⁺ with enzymes (Buchachenko et al. 2012; Buchachenko 2016; Letuta and Berdinskiy 2017). These effects impact ATP synthesis, DNA replication, and protein phosphorylation (Steiner and Ulrich 1989; Buchachenko et al. 2012, 2013; Beretta et al. 2019). Magnetic fields can also promote exopolysaccharide (EPS) production in *Bacillus cereus*. Fields of 6.0–10.0 mT enhance EPS production. At the same time, intensities up to 17.4 mT increase EPS’s negative charge, facilitating cation bio-adsorption (Xu et al. 2014; Sengupta et al. 2018). Together, these observations highlight magnetic fields as versatile modulators of bacterial physiology, with effects that depend on field type, strength, and species (Beretta et al. 2019).

### Thermosensing

**Fig. 4:**
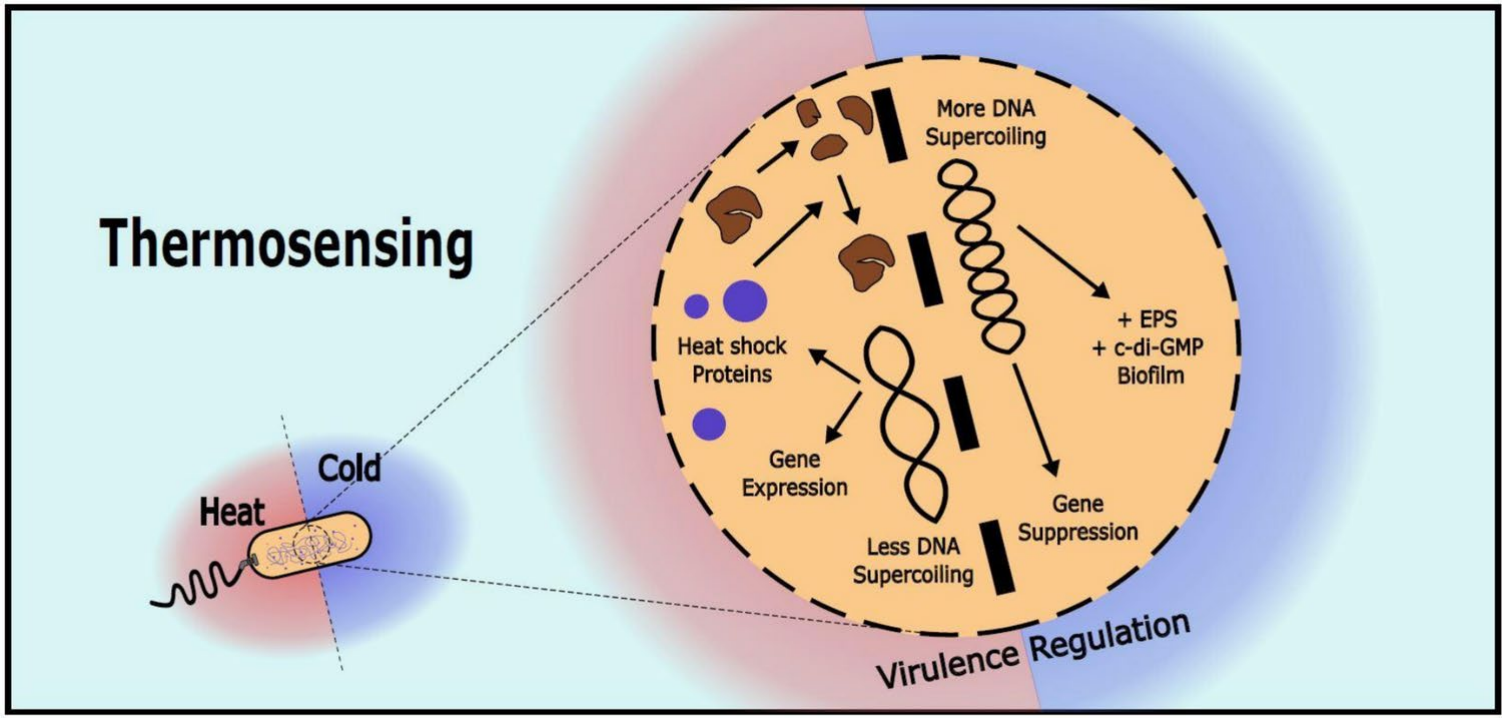
Thermosensing. Heat relaxes DNA supercoiling, exposing and activating heat-responsive genes, while heat shock proteins (e.g., RpoH) counteract protein damage. In contrast, cold increases supercoiling, condensing the DNA strand and suppressing gene expression to conserve energy and resources. It also induces the synthesis of c-di-GMP and promotes biofilm formation

At the molecular scale, thermal fluctuations may change the local structure and the reaction kinetics of molecules crucial for cellular processes, such as transcription (Moon et al. 2023). One of the main reasons why bacteria must adapt to temperature changes is because they colonize environments with temperature fluctuations. One of the most-well-studied scenarios is when bacteria colonize a host (Steinmann and Dersch 2013). Thus, it is unsurprising that temperature informs pathogens to activate virulent gene expression (Fig. 4).

Temperature is always present and determines the energetic state of molecules and their structures. Organisms sense temperature primarily through proteins that switch their functional states upon surpassing specific temperature thresholds (Moon et al. 2023). One key adaptation at high temperatures is the alteration in DNA topology. Heat relaxes DNA by enzymes that actively increase supercoiling in the direction opposite to that in which the strand is naturally supercoiled, thus exposing promoter regions and enabling RNA polymerase binding, thereby facilitating transcription of heat-responsive genes (Fig. 4) (Moon et al. 2023). This supercoiling is regulated by ATP-dependent enzymes like gyrase, activated at higher ATP levels available during heat stress, further enhancing transcriptional activity (Soini et al. 2005).

The regulation of gene expression in response to temperature also relies on specialized RNA structures known as RNA thermometers (RNATs), predominantly located in the 5′-untranslated regions (5′-UTRs). RNATs such as the Repression of Heat Shock Gene Expression (ROSE) and FourU elements maintain secondary structures at low temperatures, shielding ribosome binding sites (Moon et al. 2023). These structures melt upon heat exposure, exposing the ribosome binding sites and initiating translation of heat-responsive proteins, such as chaperones, without additional regulatory factors being needed (Abduljalil 2018). Another adaptation to high temperatures is of depleting protein-based regulatory systems known as the heat shock proteins (Hsps) like the sigma factor σ32 (RpoH). RpoH coordinates the expression of multiple chaperones and proteases that counteract protein denaturation by refolding damaged proteins or degrading irreparably damaged ones (Guisbert et al. 2004; Yura 2019). In *E. coli*, the RpoH system is also linked to a secondary heat shock response mediated by RpoE, which is a response to denatured proteins accumulating in the periplasm. RpoE induces genes related to protein folding and lipopolysaccharide synthesis, providing an adaptive advantage for membrane stability under stress (Dartigalongue et al. 2001; Rhodius et al. 2006; Moon et al. 2023).

Conversely, at low temperatures, DNA undergoes supercoiling, due to decreased gyrase efficiency, which leads to passive condensation and restricted promoter access, effectively suppressing gene expression. This structural adaptation reduces the energy expenditure associated with transcription, favoring gene silencing during cold stress (Dash et al. 2022). Cold shock proteins (CSPs) like CspA in *E. coli* function by binding RNA to promote the stabilization of single-stranded regions, aiding in maintaining translational efficiency at reduced temperatures (Ignatov and Johansson 2017; Giuliodori et al. 2023). In addition to CSPs, small RNAs such as DsrA bind the 5′-UTR of mRNA for cold shock-responsive genes, increasing their stability and translation rates under cold conditions and counteracting the overall reduction in translational activity that low temperatures induce (Moon et al. 2023).

Furthermore, certain bacterial species employ cold-regulated nucleoid-associated proteins that further modulate chromatin structure to inhibit gene transcription. These proteins can maintain a repressive chromatin state by stabilizing DNA in a highly compacted form, limiting unnecessary gene expression, and conserving resources during prolonged cold exposure (Roncarati and Scarlato 2017). On the other hand, transcription levels of several biofilm-related genes in E. coli, such as *bolA*, are increased by regulation of the sigma factor RpoS. This increase in transcription is also associated with increased EPS production, driven by the expression of CsgD, a regulator capable of sensing c-di-GMP (Moon et al. 2023). Low temperatures favor the induction of diguanylate cyclases, enzymes responsible for the production of c-di-GMP (Townsley and Yildiz 2015), which in turn activates CsgD. This regulator directly modulates the production of amyloid curli protein fibers and, indirectly, cellulose biosynthesis, both key components of EPS (Saldaña et al. 2009). Such multifaceted adaptations, which span DNA structural changes, RNA stabilization, and specific protein regulators, illustrate the robust yet nuanced bacterial response to low-temperature environments, ensuring cellular homeostasis in fluctuating conditions.

## Physical interactions within colony networks

Communication has been essential to bacteria thriving and survival since community formation provides microorganisms with many benefits in nature (Keller and Surette 2006; Dufrêne and Persat 2020; Netzker et al. 2020). In this section, we will review how bacterial communities can employ the previously highlighted physical stimuli. Furthermore, we will overview how external application of this stimuli has been used to affect collective lifestyle, motility, colony architecture, and growth (Reguera 2011; Dufrêne and Persat 2020; Maier 2021; Wong et al. 2021).

**Fig. 5:**
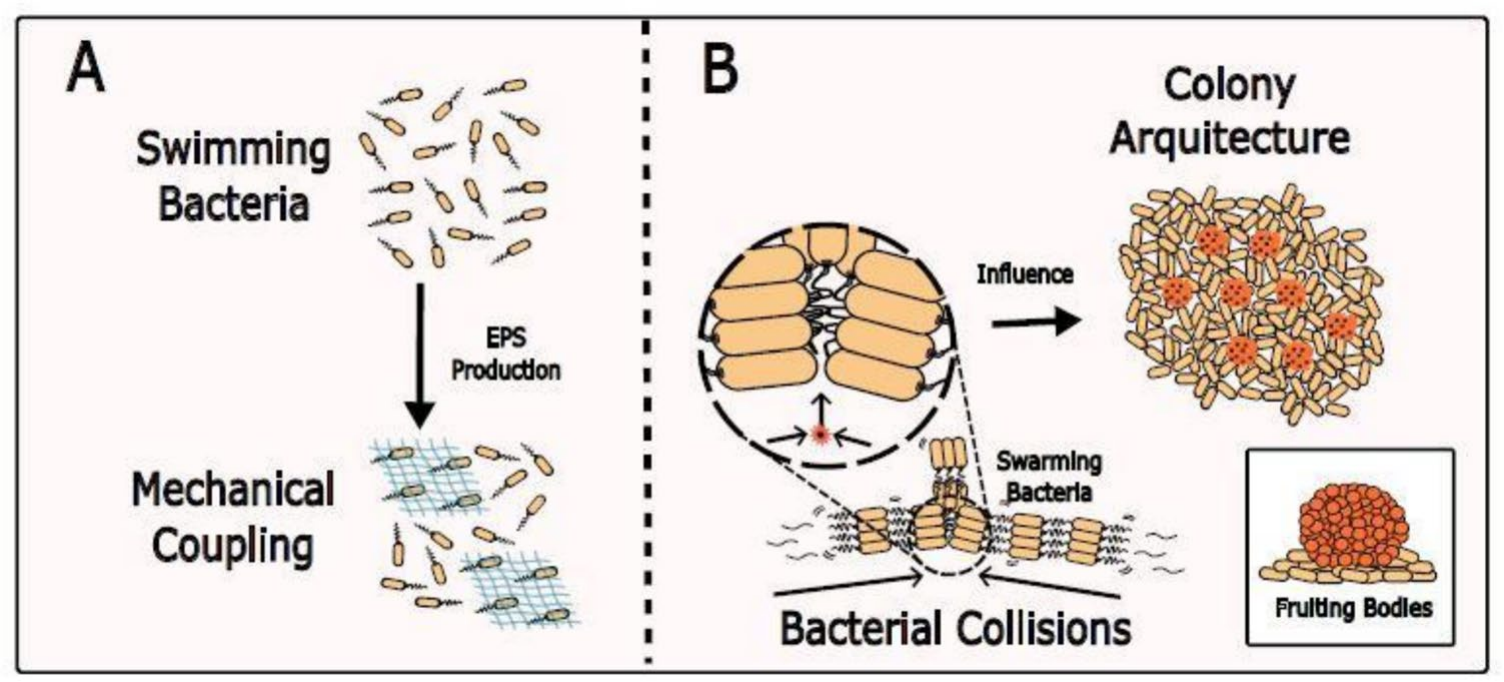
Mechanical coupling and bacterial collisions. **A** Swimming bacteria can sense the motion of distant swimmers by mechanical coupling through EPS production. **B** Collisions in bacterial swarms and direct physical contact cause cellular extrusion that can influence the assembly of the colonies in 3D and favour fruiting body formation

Mechanical interactions among bacteria happen even in dilute suspensions, before they form densely packed biofilms (Sretenovic et al. 2017). This has been demonstrated by using optical tweezers to move individual cells in dilutions of planktonic bacteria. In swimming *B. subtilis*, mechanical coupling was observed between cells spaced about 35 µm apart (Fig. 5A). The strength of the mechanical coupling is proportional to the amount of the extracellular matrix material. For higher cell densities (in excess of 109 cells per ml) of swimming *B. subtilis*, bacterial turbulence can be generated by collective swimming upon transition from random swimming to transient jet and vortex patterns (Wolgemuth 2008).

Mechanical forces among bacteria within a community also change the colony structure. Crowding in *P. aeruginosa* and *E. coli* colonies promotes buckling and multilayer formation (Takatori and Mandadapu 2020; Shimaya and Takeuchi 2022; Lama et al. 2024). In colonies of *Myxococcus xanthus*, cellular extrusion due to crowding drives the formation of fruiting bodies (Fig. 5B) (Liu et al. 2019a, b; Copenhagen et al. 2021). In *B. subtilis* swarms, bacteria crowding and collisions of motile with immotile cells promote the formation of multiple layers in the swarm which may result in enhancing biofilm expression genes and formation (Grobas et al. 2021, 2022; Worlitzer et al. 2022; Liu et al 2024c). In biofilms, extrusion forces generated by continuous growth in confined spaces lead to hollow wrinkles promoted by cell death patterns (Asally et al. 2012; Yan et al. 2019; Geisel et al. 2022). In some cases, these wrinkles are populated by motile bacteria and are believed to enhance fluid transport which might promote nutrient diffusion (Wilking et al. 2012; Rooney et al. 2020; Geisel et al. 2022). Mechanical forces within biofilms can also modulate quorum sensing and gene expression (Lin and Cheng 2019). High shear stress in *P. aeruginosa* enhances the production of quorum sensing molecules, while low shear upregulates genes involved in alginate biosynthesis, denitrification, cell shape determination, glycine betaine biosynthesis, glycerol metabolism, and tryptophan biosynthesis and downregulates genes involved in motility, phenazine biosynthesis, type VI secretion, and multidrug efflux (Dingemans et al. 2016).

**Fig. 6:**
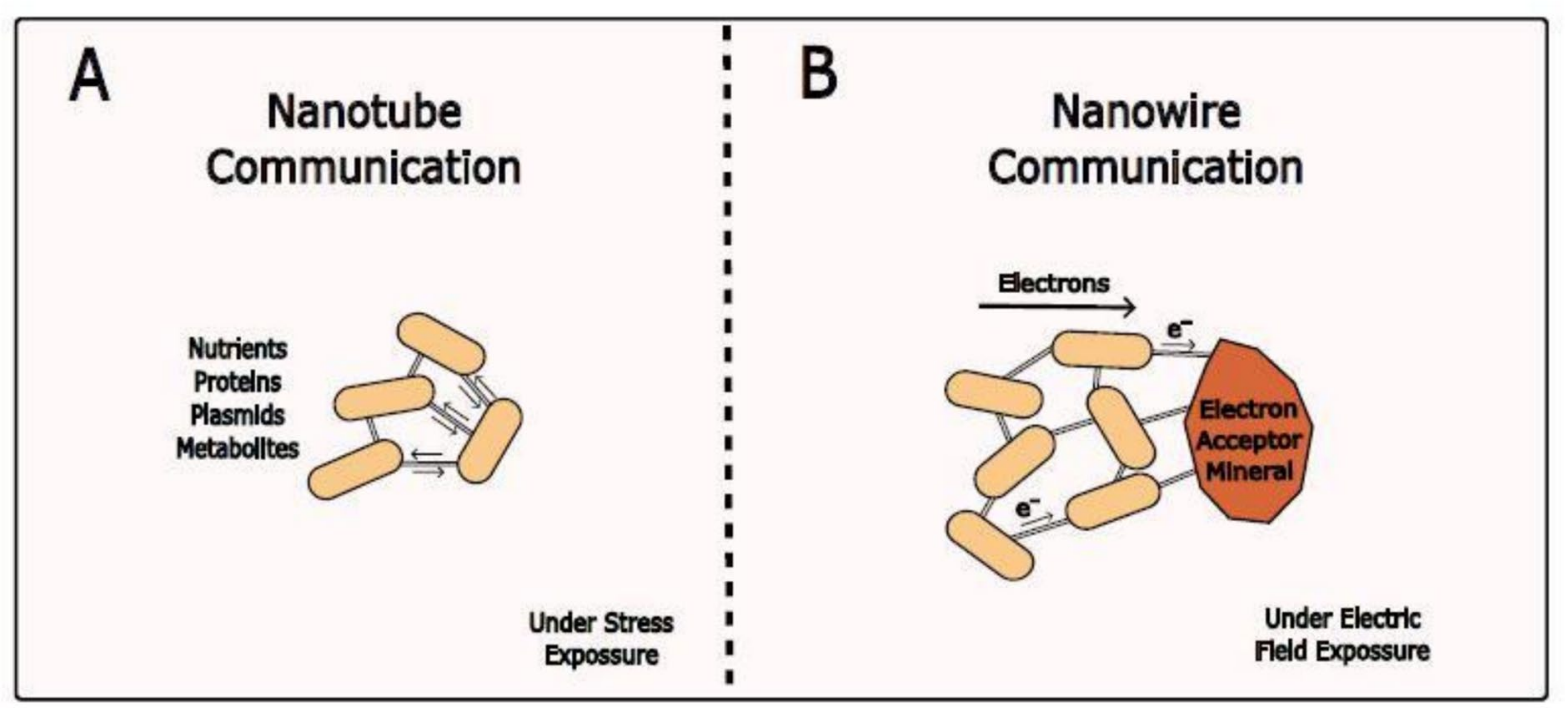
Nanotube and nanowire communication. **A** Bacteria can establish cytoplasmic connection through nanotubes, lipid-based structures that might enable the transfer of nutrients, proteins, plasmids, and metabolites. **B** Similarly, nanowires facilitate the transport of electrons outside the cell to extracellular electron acceptors (other cells or mineral substrates)

Bacteria can establish a physical connection not only through membrane-to-membrane contact but also through the use of nanotubes (Dubey et al. 2016). These lipid-based structures, varying in width and often appearing as connected vesicles, facilitate cytoplasmic connections that might enable the transfer of nutrients, proteins, plasmids, and metabolites (Fig. 6A) (Dubey and Ben-Yehuda 2011; Pande et al. 2015; Baidya et al. 2020). However, recent studies suggest that nanotube formation might be linked to cell death or stress rather than serving as a communication mechanism (Pospíšil et al. 2020). Nanotubes have been shown to be promoted by external mechanical stimulus such as nanopillar surfaces of 1 µm spacing and weight application onto *P. aeruginosa* biofilms (Cao et al. 2020). In the latter, the weight exerted a selective pressure since cells unable to produce nanotubes were wiped out and the surface was completely occupied by only nanotube producers. After the removal of weight and restoration of nutrients, these phenotypes reverted to the normal planktonic lifestyle (Ahmed et al. 2022).

**Fig. 7:**
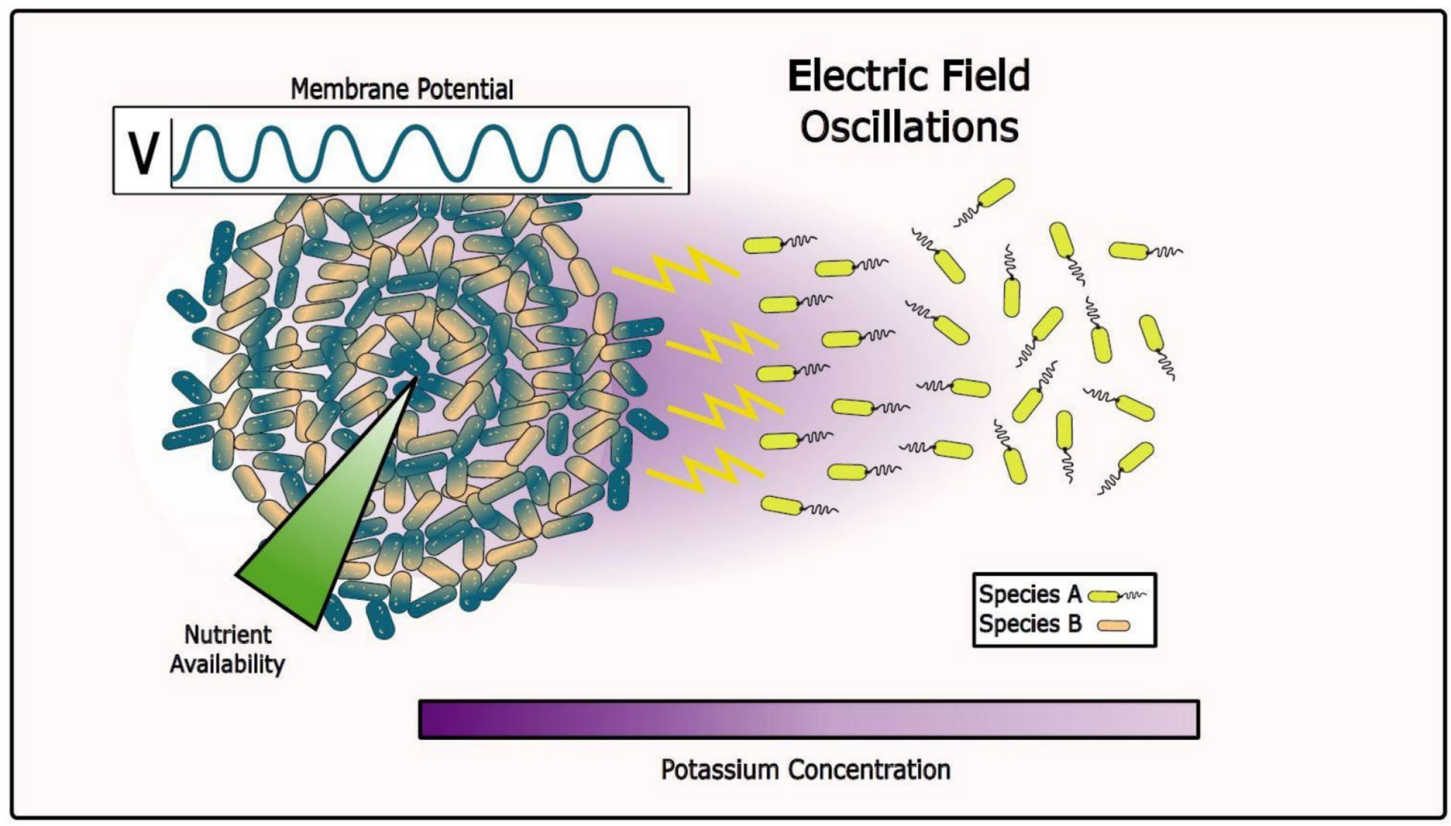
Long-range electrical communication. Electrical signaling coordinates metabolic exchange between interior and peripheral cells of biofilms. Propagating potassium waves alter the membrane potential, allowing inner cells undergoing nutrient depletion to communicate with peripheral cells. Potassium-based signaling can reach cells outside the biofilm, allowing interspecies communication

Bacterial communities can expand over distances of centimeters which would call for a longer-range form of physical communication than nanotubes and direct contact. Here, electrical communication has demonstrated to be an emergent property (Masi et al. 2015) that efficiently coordinates metabolic activities between cells at the middle and at the periphery of *B. subtilis* biofilms (Fig. 7), enabling the community to respond collectively to environmental changes (Liu et al. 2015; Prindle et al. 2015). The oscillations were demonstrated to be due to K + waves propagating at fluctuating speeds that depend on the radius of propagating front and the geometrical arrangement of the cells (Larkin et al. 2018; Blee et al. 2019; Martorelli et al. 2024). More specifically, the nutrient starved cells in the interior supply aminoacids to cells in the periphery, while peripheral cells decrease their membrane potential and provide fatty acids to the starved cells at the interior. This results in an increase of the overall antimicrobial tolerance of the biofilm, since interior cells can use those fatty acids to repair their membrane (Zhang et al. 2024). Such electrical communication has also been demonstrated to reach cells outside the biofilm and work as a form of interspecies communication (Humphries et al. 2017). *B. subtilis* biofilms can send potassium waves to individual *P. aeruginosa* cells outside the biofilm (Fig. 7). Upon reception, *P. aeruginosa* goes toward the biofilm by altering their membrane potential and changing their tumbling rate, leading to the periodic accumulation of motile cells at the biofilm edge with a frequency matching the oscillations in biofilm membrane potential, as well as an increase in the probability of motile cells becoming embedded in the biofilm (Humphries et al. 2017).

Changing the membrane potential externally has been a proven strategy to manipulate *B. subtilis* biofilms. External supply of K + ions has demonstrated to guide growth in *B. subtilis* biofilms (Dechiraju et al. 2024). The biofilm grows in the direction where potassium was supplied, with larger K + concentration leading to larger growth rates. External electrical stimulation of *B. subtilis* biofilms also promote the proliferation of motile cells and reduce the amount of matrix producing cells (Comerci et al. 2022).

The electrical counterpart to nanotubes are microbial nanowires (Wang et al. 2024), which facilitate the transport of electrons outside the cell to extracellular electron acceptors (Fig. 6B) (El-Naggar et al. 2010; Summers et al. 2010). These structures are crucial for bacteria to respire in harsh environments that lack soluble, membrane-permeable, electron acceptors such as oxygen (Wang et al. 2019). In nature, these structures are used by heterogeneous microbial communities to extend the distance of electrochemical interactions between cell-to-mineral and cell-to-cell connections (Summers et al. 2010). Nanowires can be stimulated by an applied external electric field as it has been shown in *Geobacter sulfurreducens* biofilms (Yalcin et al. 2020). The electric field stimulates production of cytochrome OmcZ nanowires with 1,000-fold higher conductivity (30 S/cm), and threefold higher stiffness (1.5 GPa), than the cytochrome OmcS nanowires that are found in nature (Yalcin et al. 2020). These discoveries present potential implications to engineer communities as living batteries.

**Fig. 8:**
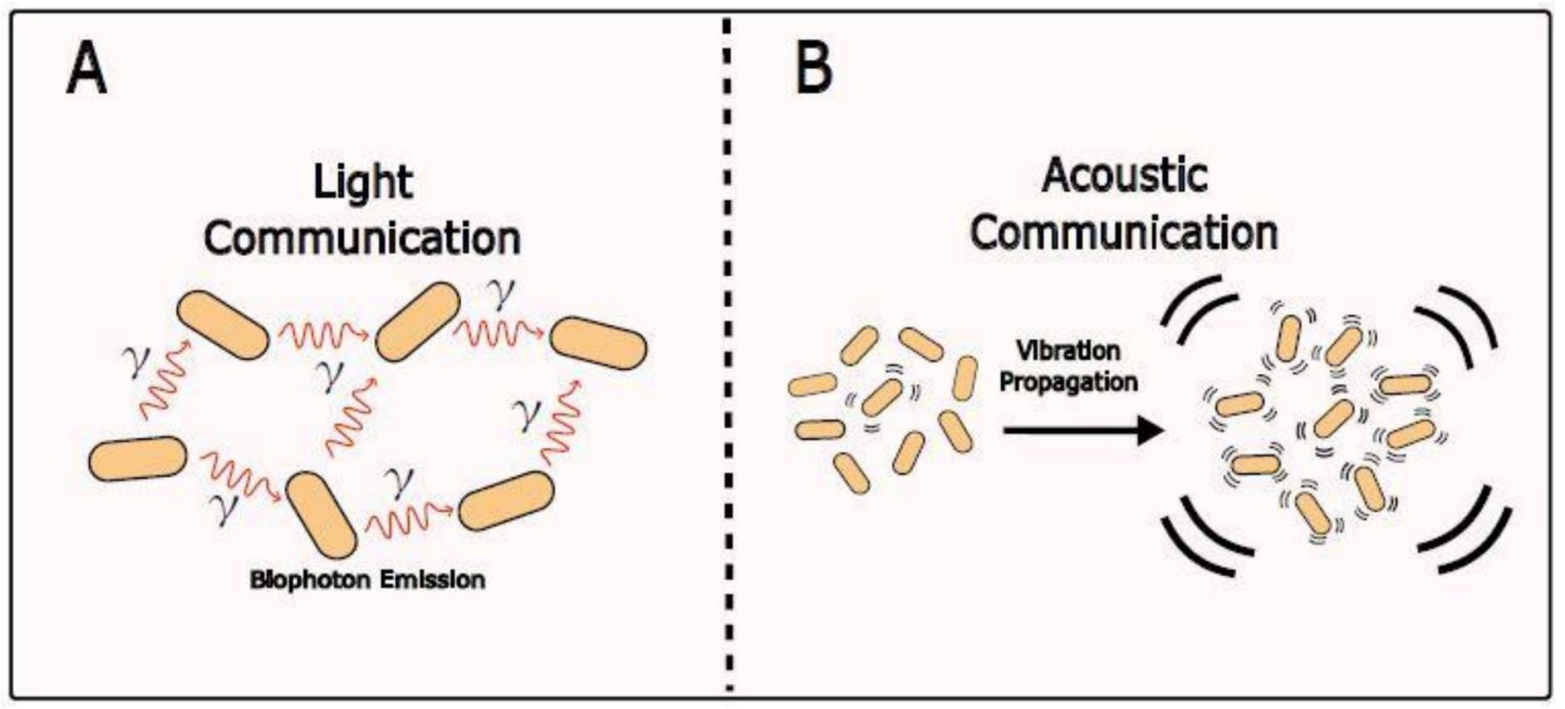
Light and acoustic communication. **A** Bacteria emit electromagnetic radiation named “biophotons” that might be affected by the presence of stress and variation of metabolic activity in the cell. **B** Bacteria can sense and emit acoustic waves that stimulate their growth and alter intracellular ions and protein content. Due to mechanical resonance, sound signals can be amplified when bacteria communicate with their surrounding cells in community situations like biofilms

Other forms of physical communication between microorganisms such as light and sound have been explored but they have been less studied (Reguera 2011; Robinson et al. 2021). Bacteria can produce electromagnetic radiation known as “biophotons” (Fig. 8A) (Tong 2024), possibly (but not yet proven) originated by oscillations of charged particles during cellular processes such as cell division (Reguera 2011). Therefore, biophotons will be affected by the presence of stresses and variation of metabolic activity in the cell. This was proven by setting up two different bacteria parallel cultures and measuring the biophotons emitted by one of them with a photomultiplier. When H_2_O_2_ was added to one of the cultures, a change in the biophotons emission spectra by the other culture was detected (Tessaro et al. 2019).

Additionally, bacteria are able to sense acoustic waves that stimulate their growth and alter intracellular ion and protein content (Sarvaiya and Kothari 2015; Azadeh et al. 2021). Acoustic waves can be emitted by other bacteria in their surroundings to a greater or lesser extent (Fig. 8B) (Matsuhashi et al. 1998). In Matsuhashi et al. (1995), it was observed that the growth of *Bacillus carboniphilus* was enhanced by the frequencies produced by external speakers and neighboring cells, even separated by 2 mm iron barriers that prevent from volatile exchange. The mechanism generating these sound waves corresponds to intracellular motions of essential cellular processes (e.g., activity of molecular motors and the cytoskeleton or chromosome packaging and replication) (Howard 2009; Ibarra et al. 2009; Müller et al. 2009), and polar protein oscillations during cell division or assembly (Huang et al. 2003; Graumann 2009). With sufficient intensity (and adequate frequency), the sound signal could reach a receptive cell with enough force to induce vibration, modulating the recipient’s metabolism by activating or deactivating DNA regulatory proteins (Norris and Hyland 1997) and thereby, modulating gene expression (Volkov and Kosevich 1991; Lisý et al. 1996; Vinayavekhin et al. 2024). It has been noticed that different species emit sound in different frequencies (Matsuhashi et al. 1998), and due to mechanical resonance, intracellular components absorb more energy from vibrations matching their own energy. Taking into account that the signal intensity is inversely proportional to the distance it travels (Gantner et al. 2006), sound signals may be received by close individuals that can amplify the signal if they are from the same species, improving communication in situations like mixed biofilms (Fig. 8B) (Reguera 2011). In particular, Garuba et al. (2021) demonstrated that specific sound frequencies modify bacterial growth and antibiotic susceptibility, consistent with the data collected by Robinson et al. (2021). Exposure of *P. aeruginosa* to a 100 Hz acoustic vibration led to enhanced biofilm formation by 0.3-fold compared to the silence control (Murphy et al. 2016). In this study, acoustic vibration of 800-Hz vibration showed increased cell density only in biofilm, but not in planktonic cells, demonstrating that the effect of sound depends on the bacteria lifestyle.

## Concluding remarks and future directions

Interactions between bacteria and their physical environment play a critical role in bacterial communication, colony expansion, and survival (Harper et al. 2023). Our review highlights the diverse physical mechanisms by which bacteria sense and respond to physical stimuli such as mechanical forces (Gordon and Wang 2019), electromagnetic radiation (Beretta et al. 2019; Wollmuth and Angert 2023) and temperature changes (Han et al. 2023). These physical cues do not only facilitate adaptation of individual cells, but also promote communication within bacterial communities, ultimately influencing colony structure and function (Liu et al. 2019a, b; Grobas et al. 2020; Black and Shaevitz 2023; Moon et al. 2023).

The integration of physical communication pathways with biochemical signaling, such as quorum sensing, adds layers of complexity and resilience to bacterial behavior, underscoring the sophisticated adaptability of microbial life (Wong et al. 2022). This interconnectedness suggests that environmental manipulation of physical cues could provide a strategic, non-lethal approach to controlling bacterial colonies, in contrast to the use of physical stimuli in sterilization. The non-destructive physical stimuli discussed in previous sections—such as sound waves, mechanical stress, and electric fields—emerge as promising tools for disrupting bacterial communication, biofilm formation, and growth, with potential applications in biomedicine and industry (Nguyen et al. 2015; Kothari et al. 2017; Palau et al. 2023; Sadeghzadeh et al. 2024). Sound waves influence microbial metabolism (Reguera 2011), and nanovibrational stimulation has been shown to reduce *E. coli* surface adhesion by altering membrane potential (Bazzoli et al. 2024). Surface acoustic waves reduce biofilms on medical devices (Hazan et al. 2006; Wang et al. 2017), and electric fields combined with shear stress inactivate bacteria in food processing (Mok et al. 2019). Mechanical stress also compromises multidrug-resistant efflux systems (Genova et al. 2019). Combining active and passive surface strategies optimizes biofilm control (Khalid et al. 2020), and hyperthermia with DNA damage approaches further boost antimicrobial effects (Alumutairi et al. 2020; Taddese et al. 2021), underscoring the need for multi-targeted strategies to avoid bacterial adaptation (Sadeghzadeh et al. 2024).

Future research should focus on refining our understanding of how these physical signals can be used more accurately to inhibit pathogenic biofilm formation or enhance the growth of beneficial bacteria (Santore 2022). In addition, advances in microfluidic and imaging technologies offer exciting potential for real-time exploration of bacterial mechanosensing and electrochemical communication (Karimi et al. 2015; Liu et al. 2024a, b, c; Wen et al. 2024). Such insights could pave the way for novel antimicrobial strategies and open avenues to exploit these fundamental aspects of bacterial physiology with promising applications in fields ranging from biomedicine to industrial bioprocessing (Mevo et al. 2021; Zhao et al. 2023).

## Data Availability

No datasets were generated or analysed during the current study.
